# The Invisible World

**Published:** 1890-04

**Authors:** 


### THE INVISIBLE WORLD.

Rev. J. Sanders Reed, rector of Trinity Church, recently delivered a striking lecture upon "The Invisible World."

"I am glad," he said, "to live in the nineteenth century, when mysteries are being lifted, and every day multiplies the analogies between science and religion, and we may hope to see the crown yet which glitters on the tripartite kingdom of science, religion and grace. Is there an invisible world? and do we enjoy our homes alone or is the air filled with spirits and aerial beings? Science says 'Yes,' and it depends upon the number of senses we have whether we agree with science. Our minds are in prisons, from which they look out through windows in the walls, and that mind which enjoys the greater outlook must see more than others. Our present inability to see angels is no argument against their existence, as what we know depends upon the number of our senses.

"The windows of the house in which we live are glazed or stained. We cannot see or hear all. The dog accompanying us through the forest scents the game of which we had no knowledge. The atmosphere is populous with particles that elude the prism and the scales, and yet they lend the sky its azure and distribute the sunbeams over the earth. Sound consists in the movement of the air and the existence of an auditory nerve. The deaf are insensible to thunder, yet it thunders.

"Negative scientific schools say that they cannot find our God any where! Does not their science teach them that there is another world which neither scalpel nor microscope can explain or explore. Scientific men know that the atmosphere is crowded with life germs, and is it too much to ask that we be permitted to believe that back of these life germs higher lives and more distinguished organisms exist. Were our ears properly attuned, we might hear the atmosphere, now silent, musical with the tread of ghostly feet, and it may all come in good time."

